# Nanoscale cutting using self-excited microcantilever

**DOI:** 10.1038/s41598-021-04085-y

**Published:** 2022-01-12

**Authors:** Rui Yang, Ichiro Ogura, ZhenYan Jiang, LinJun An, Kiwamu Ashida, Hiroshi Yabuno

**Affiliations:** 1 Graduate School of Systems and Information Engineering, University of Tsukuba, 1-1-1, Tennodai Tsukuba City, 305-8573 Japan; 2 Advanced Manufacturing Research Institute, National Institute of Advanced Industrial Science and Technology, 1-2-1, Namiki, Tsukuba, 305-0044 Japan

**Keywords:** Engineering, Nanoscience and technology

## Abstract

The application of self-excitation is proposed to improve the efficiency of the nanoscale cutting procedure based on use of a microcantilever in atomic force microscopy. The microcantilever shape is redesigned so that it can be used to produce vibration amplitudes with sufficient magnitudes to enable the excitation force applied by an actuator to be transferred efficiently to the tip of the microcantilever for the cutting process. A diamond abrasive that is set on the tip is also fabricated using a focused ion beam technique to improve the cutting effect. The natural frequency of the microcantilever is modulated based on the pressing load. Under conventional external excitation conditions, to maintain the microcantilever in its resonant state, it is necessary to vary the excitation frequency in accordance with the modulation. In this study, rather than using external excitation, the self-excitation cutting method is proposed to overcome this difficulty. The self-excited oscillation is produced by appropriate setting of the phase difference between the deflection signal of the microcantilever and the feedback signal for the actuator. In addition, it is demonstrated experimentally that the change in the phase difference enables us to control the amplitude of the self-excitation. As a result, control of the cutting depth is achieved via changes in the phase difference.

## Introduction

Various types of micromachining technology have been established for use in microelectromechanical systems (MEMS) manufacture^1^. Wet and dry etching processes^2,3^, which enable removal of materials for silicon bulk micromachining, are excessively dependent on variations in the concentrations of the rare chemicals used and the extreme environmental created^4,5^. Laser processing^6^ and focused ion beam processing^7^ techniques, which ablate materials using focused beams of light or ions, respectively, is limited in processing accuracy by the divergence of the beam focal point described by the wave theory of light^8^ and the alteration in the target layer due to heating by the incident beam^9^. Also, these micromachining technologies are unsuitable for nanoscale precision fabrication of structures with non-flat surfaces. However, this problem was previously solved using cutting methods that relied on a force control system that could cut inclined or curved surfaces to the same depth^10–12^.

Atomic force microscopy (AFM)^13^, which has been used for nanoscale imaging of surfaces, has also been used as a force control system for nanomachining to cut hole of approximately 10 nm depth in single-crystal silicon^14^. The control of the distance between the microcantilever tip and the sample surface in the AFM system was appropriate for application of a constant force during the cutting process. In addition, a microcantilever has been equipped with a diamond on the tip, which acted as a useful cutting tool^15^. The required hole has been achieved to be cut to greater depths by increasing the pressing load set on the tip of the microcantilever^16^. Nanomachining technology using the microcantilever in the AFM system has been demonstrated to be a low-cost, high-precision method for cutting of various materials into nanodots, nanolines, and even two-dimensional and three-dimensional structures^17^. Those previous methods have limitations in improvement of the process efficiency. On the other hand, various types of vibration-assisted machining method have been proposed for enhancing process efficiency, where either external excitation or self-excitation is generally used^18,19^. This study intends to propose a new practical nanomachining technology and discuss the theoretic support by the fusion of the vibration-assistance.

In this research, the shape of the microcantilever is redesigned by adding a base step to produce the required magnitudes of the vibration amplitudes. A diamond abrasive is set on the tip and fabricated sharper using focused ion beam (FIB) technology. Because the natural frequency of the microcantilever is modulated based on the different pressing loads applied, the microcantilever cannot be maintained in the resonant state at a constant excitation frequency under external excitation. To overcome this difficulty, a new vibrational cutting method is proposed that uses a self-excited microcantilever. Through appropriate setting of the phase difference between the microcantilever deflection signal and the feedback signal for the actuator, self-excited oscillation in the microcantilever is produced experimentally and the resonance mechanism is then clarified theoretically. Furthermore, it is demonstrated experimentally that variation of the phase difference enables control of the amplitude of the self-excitation. As a result, variable cutting depths are cut by changing the phase difference appropriately. The proposed nanoscale cutting using a self-excited microcantilever can be expected to improve the accuracy of the measurement for the 3D characterization of the mechanical response of the sample surface in Tomographic AFM^20^ by controlling the amplitude of the microcantilever by phase modulation in air and improve the limit of Scalpel AFM^21^ by causing a deformation in appropriate depth under a constant light pressing load. Also, there are other beneficial functions such as controlling the coefficient of friction^22^, modifying the optical properties^23^ and adjusting the surface wettability^24^.

## Method and analysis

### AFM application to nanoscale cutting

**Figure 1:**
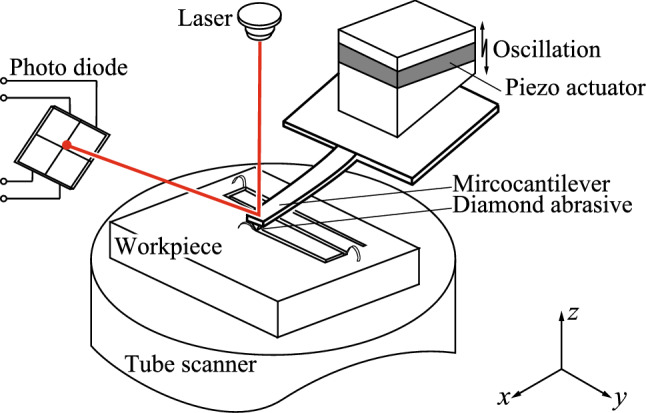
Schematic diagram of AFM system applied to nanoscale vibration cutting method. The system includes a workpiece, a microcantilever with a diamond set on its tip, a piezo actuator to enable the microcantilever oscillation, an optical lever consisting of a laser and a quadrant photodiode to measure the deflection of the microcantilever tip and a tube scanner to move the workpiece in the *x*-*y* plane and vary the distance between the microcantilever and the workpiece in the *z*-direction.

Figure 1 shows the AFM system used for the nanoscale cutting operations. The microcantilever is connected to the bottom of the piezo actuator through the holder. The piezo actuator is then charged to cause the microcantilever to oscillate. The optical lever, which consists of a laser and a quadrant photodiode, measures the deflection of the microcantilever tip. The tube scanner is charged to move the workpiece within the *x*-*y* plane and also performs positional control in the *z*-direction to maintain a constant pressing load during cutting.

### Redesigned microcantilever and diamond abrasive

**Figure 2:**
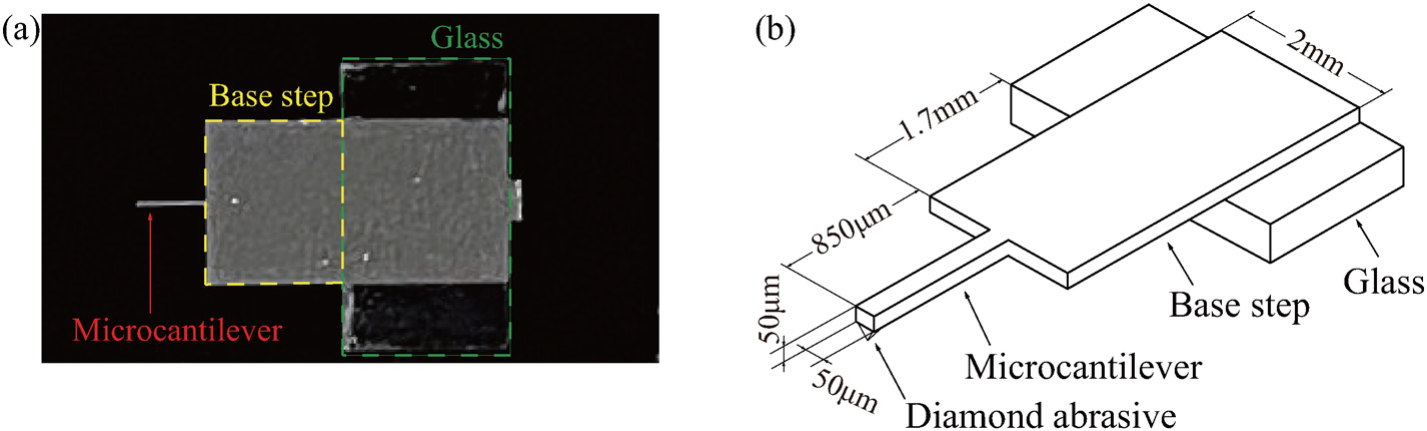
Photo (**a**) and Schematic diagram (**b**) of redesigned microcantilever. The length, width and thickness of the base step are 1.7 mm, 2 mm and 50 $\upmu$m, respectively. The length, width and thickness of the microcantilever are 850 $\upmu$m, 50 $\upmu$m and 50 $\upmu$m, respectively.

By setting the same pressing load on the microcantilever tip, the holes were cut to the same depth regardless of the application of the excitation because the response amplitude at the microcantilever tip was too small, even in the resonant state. Therefore, to produce a sufficient response amplitude magnitude to ensure that the excitation force can be transferred efficiently to the tip, the microcantilever shape is redesigned which is shown in Fig. 2a. In this design, a base step is connected to the microcantilever. The length, width and thickness of this base step are 1.7 mm, 2 mm and 50 $\upmu$m, respectively. The length, width and thickness of the microcantilever are 850 $\upmu$m, 50 $\upmu$m and 50 $\upmu$m, respectively, as shown in Fig. 2b.

**Figure 3:**
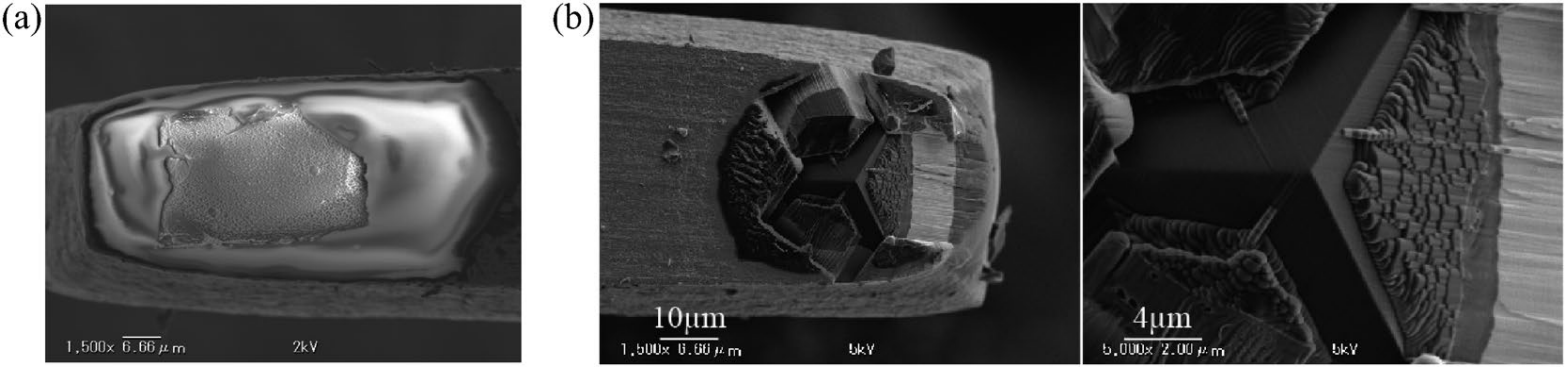
SEM image of the original diamond abrasive on the tip of the microcantilever (**a**). SEM image of the diamond abrasive fabricated by focused ion beam technique (**b**). The fabricating was performed twice, one was rough processing with an ion beam in a diameter of 500 nm and the other was precise processing with an ion beam in a diameter of 200 nm. The angle of the fabricated diamond is 90 degrees; this angle is formed by the sides and the triangular faces at the profile vertex.

The microcantilever with the original diamond could not cut a hole, because the original diamond is random and not sharp, as shown in Fig. 3a. Therefore, to achieve the cutting effect, the diamond was fabricated into a tetrahedron shape using FIB technique which can sputter the excess material of the diamond by the gallium (Ga+) primary ion beam^25,26^, as shown in Fig. 3b. The angle of the fabricated diamond is 90 degrees; this angle is formed by the sides and the triangular faces at the profile vertex. The fabricating was performed twice to ensure the high-precision cutting; the first fabricating was rough processing with an ion beam in a diameter of 500 nm to scrape off excess material and the second one was precise processing with an ion beam in a diameter of 200 nm to achieve three relatively smooth faces. The height of the fabricated diamond can be calculated graphically about 5 $\upmu$m, the aspect ratio in height of the fabricated diamond and the microcantilever is about 0.1. Hereafter, this redesigned microcantilever is referred to as the microcantilever.

### Natural frequency of microcantilever

The first natural frequency of the cantilever is calculated^27^ to be

1 $$\begin{aligned} f _{1}=\frac{{\lambda _{1}}^{2}}{2\pi l^{2}}{\sqrt{\frac{EI}{\rho A}}}, \end{aligned}$$

where $\lambda _{1}$, *l*, $\rho$ and *A* denote the first-order eigenvalue, the length, the density and the cross-sectional area of the microcantilever, respectively. *E* and *I* are the Young’s modulus and the moment of inertia of the area, respectively, as summarized in Table 1. Using Eq. (1), the first natural frequencies of the microcantilever and the base step are calculated to be 55.97 kHz and 14.06 kHz, respectively.

**Table 1:** Parameters of the microcantilever.

Description	Symbol	Value	Unit
First-order eigenvalue	$\lambda _{1}$	1.875	
Young’s modulus	*E*	1.93 $\times$ $10^{5}$	$\mathrm{Pa}$
Density	$\rho$	7.93 $\times$ $10^{-13}$	${{\mathrm{kg}}/{\mathrm{m}}^{3}}$
Length of microcantilever	${l}_{m}$	0.85 $\times$ $10^{-3}$	m
Length of base step	${l}_{b}$	1.7 $\times$ ${10^{-3}}$	m
Moment of inertia of area of microcantilever	${I}_{m}$	0.52 $\times$ $10^{-18}$	${{\mathrm{m}}^{4}}$
Moment of inertia of area of base step	${I}_{b}$	0.21 $\times$ $10^{-16}$	${{\mathrm{m}}^{4}}$
Cross-sectional area of microcantilever	${A}_{m}$	0.25 $\times$ $10^{-8}$	${{\mathrm{m}}^{2}}$
Cross-sectional area of base step	${A}_{b}$	0.1 $\times$ $10^{-6}$	${{\mathrm{m}}^{2}}$

**Figure 4:**
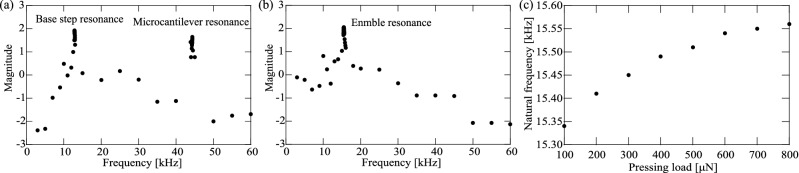
Frequency response curves with a log scale (**a**) in air and (**b**) under a 400 $\upmu$N pressing load. The first natural frequencies of the microcantilever and the base step in air were experimentally measured to be 44.35 kHz and 12.88 kHz, respectively. The first natural frequency under a 400 $\upmu$N pressing load was experimentally measured to be 15.49 kHz. Natural frequency of microcantilever under various pressing loads (**c**) with application of external excitation.

Figure 4a,b show the frequency response curves with a log scale under external excitation in air and under a pressing load of 400 $\upmu$ N, respectively. The first natural frequencies of the microcantilever and the base step in air were measured to be 44.35 kHz and 12.88 kHz, respectively. These values show close agreement with the theoretical frequencies. The first natural frequency of the microcantilever is 15.49 kHz under a pressing load of 400 $\upmu$N. The response amplitude is of sufficient magnitude to transfer the excitation force to the tip effectively. According to Fig. 4a,b, there is still a resonance for the microcantilever under the contact condition. The first peak in the response frequency curve with respect to the base step changes very little under the situation that the boundary condition at the microcantilever tip is changed from free to contact. On the other hand, the second peak in the response frequency curve with respect to the microcantilever changes to a much higher frequency. However, the peak is hard to detect because the amplitude of the microcantilever is highly damped under the contact condition. Therefore, the peak in the response frequency curve with respect to the microcantilever under the contact condition cannot be seen in Fig. 4b. Also, the resonance of the microcantilever is sensitive to the pressing loads at the tip of the microcantilever as shown in Fig. 4c. The natural frequency can be easily changed depending on the pressing load, the microcantilever is not maintained in its resonant state under the the external excitation. The magnitude of the microcantilever amplitude under the contact condition shown in Fig. 4b is larger than that shown in Fig. 4a. The reason is theoretically investigated in Appendix A.

### Cutting method using external excitation

**Figure 5:**
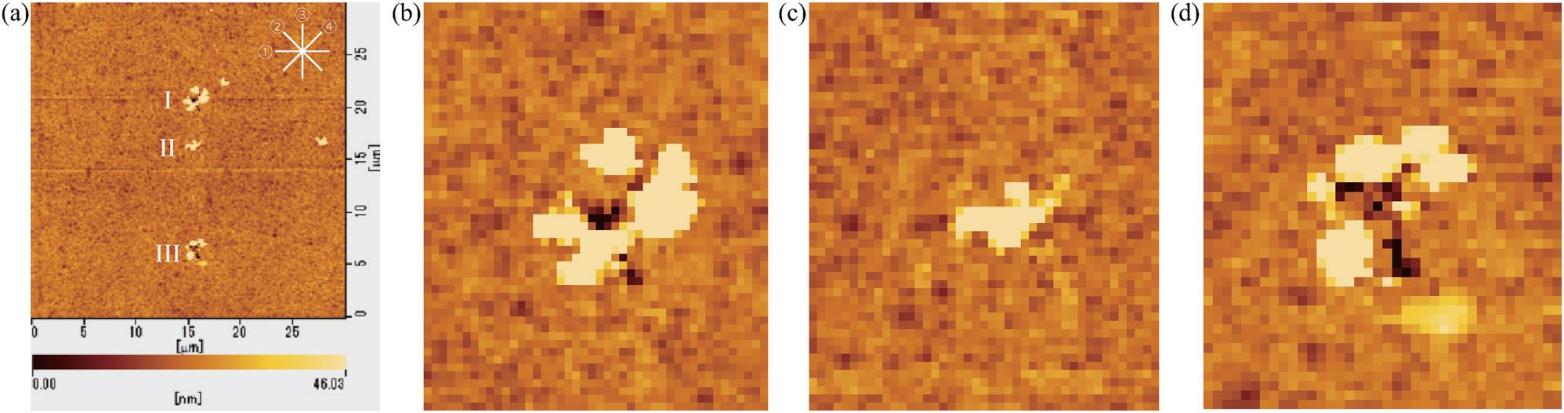
AFM image (**a**) shows three cutting results marked as I, II and III with the external excitation cutting method. The hole I was first cut under application of a 200 $\upmu$N pressing load at an excitation frequency of 15.41 kHz which is the natural frequency under the pressing load of 200 $\upmu$N. The cutting result II was secondly made under application of a 400 $\upmu$N pressing load at an excitation frequency of 15.41 kHz. The hole III was finally cut under application of a 400 $\upmu$N pressing load at an excitation frequency of 15.49 kHz which is the natural frequency under the pressing load of 400 $\upmu$N. Four lines $\textcircled {1}$–$\textcircled {4}$ in the upper corner are to indicate that the locations taken for the sectional views of three cutting results shown in Fig. 1 in Appendix B and the intersecting sectional views are centered on all cutting results. (**b**–**d**) show the magnified depiction for the AFM image of each cutting result.

All experimental results were measured in air condition by the tapping mode in AFM. The spatial resolution of AFM in *z*-direction is 0.1 nm. The commercial AFM cantilever (SII SI-DF20; SEIKO Instruments Inc.) is made of silicon. The tip radius of the AFM cantilever is less than 10nm and the specified spring constant of the AFM cantilever is 17 N/m. In the previous experiment, there was no indentation under 400 $\upmu$N pressing load without vibrational excitation. Three cutting results were made in sequence and marked as I, II and III using the same microcantilever under the external excitation, as shown in Fig. 5a. The cutting results I and II were cut under application of 200 $\upmu$N and 400 $\upmu$N pressing loads, respectively, at an excitation frequency of 15.41 kHz which is the natural frequency under the pressing load of 200 $\upmu$N. The cutting result III was cut under application of a 400 $\upmu$N pressing load at an excitation frequency of 15.49 kHz which is the natural frequency under the pressing load of 400 $\upmu$N. Four lines $\textcircled {1}$–$\textcircled {4}$ in the upper corner of Fig. 5a are to indicate the locations taken for the sectional views of three cutting results which are shown in Fig. 1a–c in Appendix B, respectively. The intersecting sectional views are centered on all cutting results. Figure 5b–d show the magnified depiction for the AFM image of each cutting result.

In this study, to compare with the case without excitation directly, the deepest value of holes or grooves is used to quantitatively evaluate the advantages of the vibrational cutting methods. The depth of the cutting results reflects the difference between the average of the baseline surface height and the depth of the hole. The error bar reflects the largest and smallest values of the baseline surface height. As indicated by the sectional views shown in Fig. 1b, the cutting result II was not cut into hole. We infer that the bump in the cutting result II is made by the chip adhered to the diamond in the first cutting experiment in which the microcantilever was externally excited with the resonance state under 200 $\upmu$N pressing load. The hypothesis is considered as follows. During the first cutting experiment, the hole I was cut and the chip was adhered to the diamond. During the second cutting experiment, the microcantilever could not cut hole when it was not resonated with its natural frequency, the chip dropped and adhered on the surface of the workpiece. After the second cutting experiment, there was not chip adhered to the diamond. Therefore, there was not a bump in the third cutting experiment in which the hole III was cut with the resonance state of the microcantilever.

**Figure 6:**
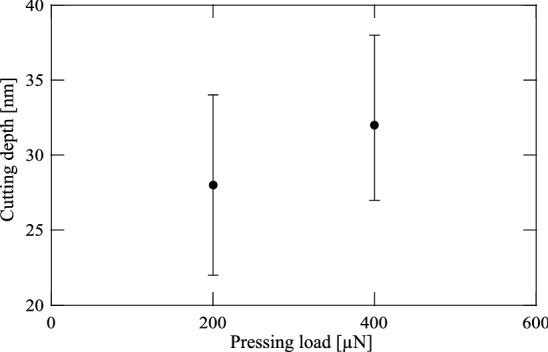
Depths for the holes I and III are the average values for all sectional views for each hole. The hole I was cut under application of a 200 $\upmu$N pressing load at an excitation frequency of 15.41 kHz which is the natural frequency under the pressing load of 200 $\upmu$N. The hole III was cut under application of a 400 $\upmu$N pressing load at an excitation frequency of 15.49 kHz which is the natural frequency under the pressing load of 400 $\upmu$N.

Tables 1 and 2 in Appendix B summarize the depth of the holes I and III calculated by each sectional view. Table 3 in Appendix B summarises the cutting condition for each cutting hole and the depth of each cutting hole calculated in average for all sectional views. Figure 6 compares the depth of the cutting holes I and III. Comparisons of the cutting results I and II show that the hole cannot be cut to be deeper when the microcantilever is not in its resonant state. Comparisons of the cutting results I and III in Fig. 6 reveal that a deeper hole can be cut if the microcantilever remains in the resonant state, even if the natural frequency is changed under the larger pressing load. Consequently, in the case of vibrational cutting using external excitation, it is necessary to measure the natural frequency modulation depending on each pressing load in advance to tune the excitation frequency to be the natural frequency during the process. To overcome this difficulty, a self-excitation cutting method is proposed in this research that can maintain the microcantilever in its resonant state independently of the pressing load during cutting without the need for tuning the excitation frequency.

### Analytical model of self-excited microcantilever

**Figure 7:**
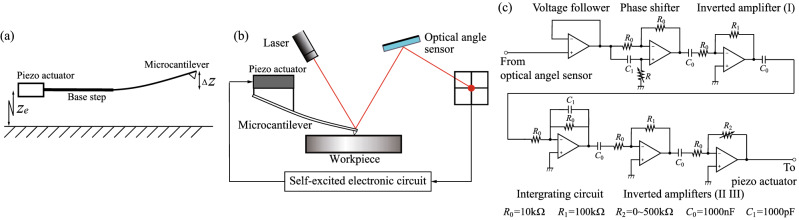
Schematic diagram of self-excited microcantilever (**a**). Schematic diagram of the self-excited system (**b**) includes a workpiece, a microcantilever, a piezo actuator, a self-excited electronic circuit and an optical lever that consists of a laser and a photodiode. Self-excited electronic circuit (**c**) includes a voltage follower, a phase shifter, an integrating circuit and three inverting amplifiers.

Self-excitation of the microcantilever used in AFM has been produced via linear feedback control^28,29^. We therefore introduce a discretized model with a single-degree-of-freedom for the microcantilever system with the base step, as shown in Fig. 7a.

The dynamics of the proposed self-excited microcantilever in air are governed by

2 $$\begin{aligned} m{(\ddot{z}_{e}+\Delta {\ddot{z}})}+c {\Delta {\dot{z}}}+k \Delta z=0, \end{aligned}$$

where *m*, *c* and *k* are the mass, the damping and the stiffness of the microcantilever, respectively. $\Delta z$ represents the displacement of microcantilever tip. $z_{e}$ is the applied displacement for self-excitation. We set the integral feedback to be

3 $$\begin{aligned} z_{e}=-k_{lin} \int {\Delta z}dt, \end{aligned}$$

where $k_{lin}$ is the linear feedback gain. Substitution of Eq. (3) into Eq. (2) yields

4 $$\begin{aligned} m{\Delta \ddot{z}}+(c-c_{lin}){\Delta {\dot{z}}}+k\Delta z=0, \end{aligned}$$

where $c_{lin}={m}{k_{lin}}$. We produce the self-excited oscillation using negative damping. Therefore, the feedback gain $c_{lin}$ is set to ensure that $c-c_{lin}$ is negative. In practice, the equivalent beam displacement $\Delta {z}$ is measured using an optical lever and the displacement $z_{e}$ is applied by a piezo actuator, as shown in Fig. 7b. The input signal to the piezo actuator is generated by the feedback circuit, as shown in Fig. 7c, where the input and output are the output signal from the optical lever and the input signal to the piezo actuator, respectively. Hereafter, this circuit is called the self-excited electronic circuit.

### Comparison between vibrational cutting methods using external excitation and self-excitation

**Figure 8:**
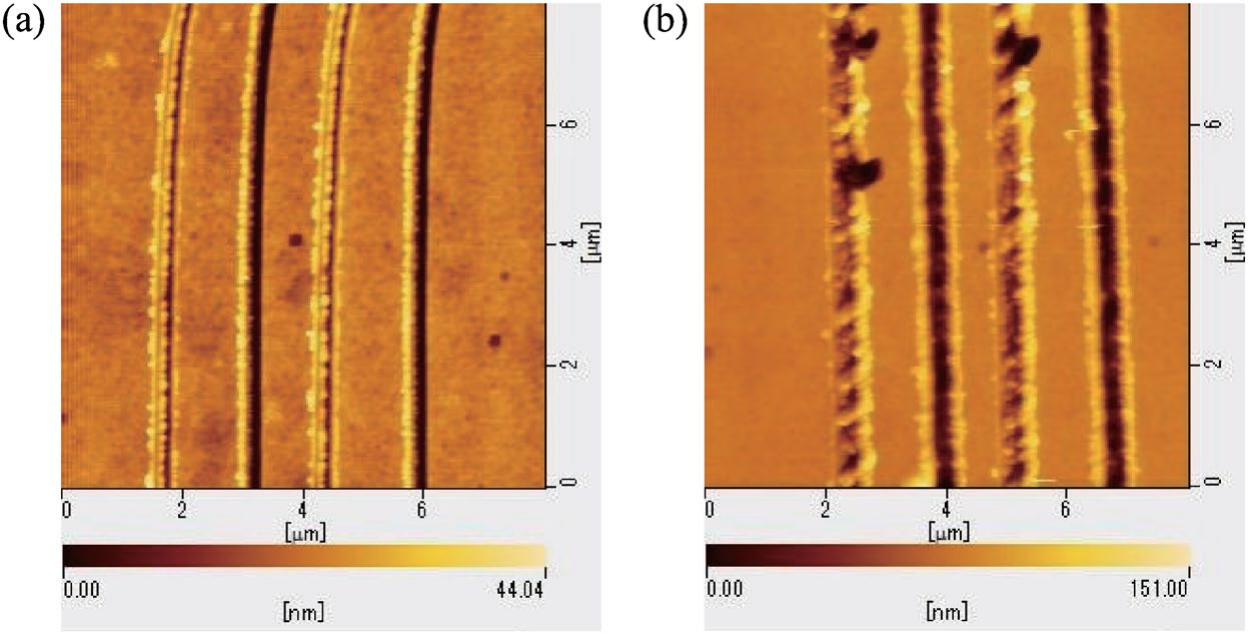
AFM image (**a**) and (**b**) show the grooves cut under 400 $\upmu$N pressing load with the external excitation cutting method and the self-excitation cutting method, respectively.

**Figure 9:**
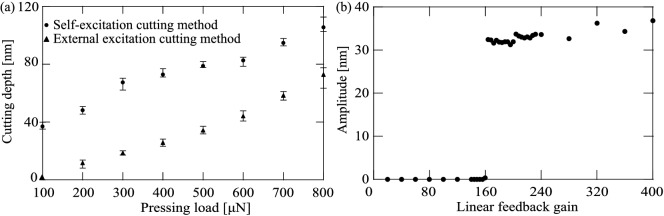
Depths of grooves cut under each pressing load with two types of cutting method (**a**). The triangles and the dots represent the depths using the external excitation cutting method and the self-excitation cutting method, respectively. Change in amplitude caused by controlling linear feedback gain $k_{lin}$ [1/s] under application of a 400 $\upmu$N pressing load (**b**).

We now compare the two types of vibrational cutting method using external excitation and self-excitation. In the external excitation cutting method, the microcantilever is excited at its natural frequency measured under a 400 $\upmu$N pressing load with a 20 Vp-p sinusoidal signal. In the self-excitation cutting method, the input of the piezo actuator is adjusted to be 20 Vp-p via linear feedback control. The depths of the grooves cut under each pressing load are shown in Fig. 9a, where the triangles and the dots represent the cutting depths achieved when using the external excitation cutting method and the self-excitation cutting method, respectively. The AFM images of the grooves cut under 400 $\upmu$N pressing loads with the two types of vibrational cutting method are shown in Fig. 8a,b, respectively. Because the self-excitation cutting method maintains the resonant state automatically under any pressing load, the scratches are cut more deeply under each pressing load than in the external excitation case. Therefore, it is demonstrated experimentally that the self-excitation cutting method offers higher efficiency for nanoscale cutting regardless of the pressing load.

### Amplitude change using linear feedback control

To cut a hole with variable depth, amplitude control of the microcantilever is required. The solution to Eq. (4) can easily be expressed as

5 $$\begin{aligned} {\Delta {z}}(t)=e^{\sigma t}{(a_{1} \cos \omega _{d} t + a_{2} \sin \omega _{d} t)}. \end{aligned}$$

where $\sigma = (c_{lin}-c) / 2 m$ and $\omega _{d} = {\sqrt{4 m k - (c_{lin}-c)}} / 2 m$. Here, $a_{1}$ and $a_{2}$ are constants that are determined by the initial conditions. The amplitude increases to infinity in theory in the case where the negative damping $\sigma >0$. However, in the experimental results, the response amplitude is kept constant. It is well known that the self-excited oscillator can only maintain a constant amplitude when external nonlinear effects act on the system^30^. We do consider methods to change the amplitude experimentally, but the theoretical investigation required to determine the source of the nonlinearity for the constant amplitude will form part of a future work.

Figure 9b shows the change in the response amplitude under application of a 400 $\upmu$N pressing load caused by variation of the feedback gain. In the case where the linear feedback gain $k_{lin}$ is above approximately 160 [1/s], the self-excited oscillation is produced. Even if the linear feedback gain is increased, the response amplitude does not change greatly. Variation of the feedback gain cannot be used for the amplitude control required to change the depth of the cut hole. Therefore, another way must be found to change the magnitude of the amplitude.

## Results and discussion

### Theoretical analysis of self-excitation using phase difference

A method based on linear feedback control was proposed in previous section. However, this method is not suitable for amplitude control. In this section, we theoretically consider another method to produce the self-excitation that involves setting the linear feedback for negative damping and shifting the phase difference between the microcantilever deflection signal and the feedback signal for amplitude control. The experimental verification of this method is discussed in the next section.

**Figure 10:**
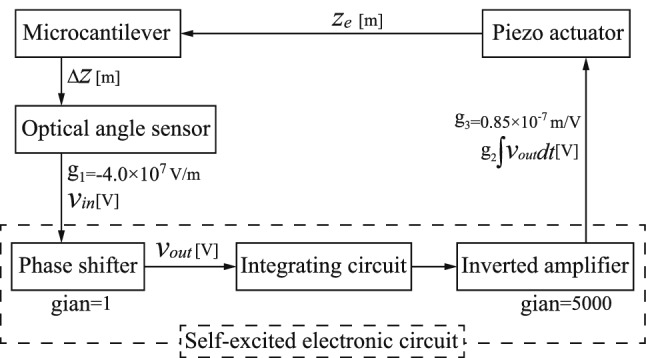
Block diagram of the self-excited system.

Figure 10 shows a diagram of the entire self-excited vibration system. To perform a theoretical analysis of the process of self-excitation by phase modulation, we rewrite $\Delta z$ and $z_{e}$ as the input and output voltages of the phase shifter $v_{in}$ and $v_{out}$ using the relationships ${v_{in}}={g}_{1}{\Delta z}$ and ${z_{e}}={g}_{2}{{g}_{3}}\int {v_{out}}dt$, respectively, where ${g}_{1}$ is the sensitivity of the optical lever, ${g}_{2}$ (5000 [1/s]) is the product of the gains of the inverted amplifier (I), the integration circuit and the inverted amplifiers (II) (III), which are 10, 1/s, 10 and 50, respectively, ${g}_{3}$ (0.85 ${\times }{10^{-7}}$ m/V) is the piezoelectric coefficient of the piezo actuator that is experimentally determined subsequently in accordance with the experimental value. Consequently, the equation for the self-excitation of the microcantilever and the differential equation for the phase shifter in the self-excited electronic circuit are rewritten as

6 $$\begin{aligned} {\left\{ \begin{array}{ll} m{\ddot{v}_{in}}+c{{\dot{v}}_{in}}+k{v_{in}}= -{{g}_{1}}{{g}_{2}}{{g}_{3}}{m}{{\dot{v}}_{out}},\\ {C_{1}}{R}{{\dot{v}}_{in}} - {v_{in}}={C_{1}}{R}{{\dot{v}}_{out}} + {v_{out}}, \end{array}\right. } \end{aligned}$$

where $C_{1}$ and *R* are the values of the capacitance and the resistance in the phase shifter, respectively, as shown in Fig. 7c. Equation (6) is nondimensionalized using the dimensionless time $t^{*}$ = $\sqrt{k/m}$ *t* as follows

7 $$\begin{aligned} {\left\{ \begin{array}{ll} {\ddot{v}_{in}}+{\gamma }{{\dot{v}}_{in}}+{v_{in}}=-{\beta }{{\dot{v}}_{out}}, \\ {\alpha }{{\dot{v}}_{in}}-{v_{in}}={\alpha }{{\dot{v}}_{out}}+{v_{out}}, \end{array}\right. } \end{aligned}$$

where

8 $$\begin{aligned} {\alpha } = {C_{1}}R\sqrt{\frac{k}{m}}, \quad {\beta } = {{g}_{1}}{{g}_{2}}{{g}_{3}}\sqrt{\frac{m}{k}}, \quad {\gamma } = \frac{c}{\sqrt{mk}}. \end{aligned}$$

Equation (7) is then rewritten into matrix form as

9 $$\begin{aligned} \frac{\mathrm {d}}{\mathrm {d} t^{*}} \left[ \begin{array}{c} v_{in} \\ {{\dot{v}}_{in}} \\ v_{out} \end{array} \right] =A \left[ \begin{array}{c} v_{in} \\ {{\dot{v}}_{in}} \\ v_{out} \end{array} \right] , \end{aligned}$$

where

10 $$\begin{aligned} A = \left[ \begin{array}{ccc} 0 &{} 1 &{} 0 \\ \frac{\beta }{\alpha }-1 &{} -\gamma -\beta &{} \frac{\beta }{\alpha } \\ -\frac{1}{\alpha } &{} 1 &{} -\frac{1}{\alpha } \end{array} \right] . \end{aligned}$$

Using eigenvalue analysis, we investigate the possibility that self-excitation can be produced by varying the dimensionless parameter $\alpha$, which is related to the value of the resistance *R* in the phase shifter. The characteristic equation of the matrix in Eq. (10) is derived as

11 $$\begin{aligned} \lambda ^{3} + \left( \beta + \gamma + \frac{1}{\alpha }\right) \lambda ^{2} + \left( 1 - \frac{\beta - \gamma }{\alpha }\right) \lambda + \frac{1}{\alpha } = 0. \end{aligned}$$

The system parameters are summarized in Table 2. The piezoelectric coefficient of the piezo actuator is identified experimentally using eigenvalue analysis.

**Table 2:** Parameters of the microcantilever and the self-excited system.

Description	Symbol	Value	Unit
Mass	*m*	1.37 $\times$ $10^{-6}$	${\mathrm{kg}}$
Stiffness	*k*	260	${\mathrm{N/m}}$
Damping coefficient	*c*	0.12 $\times$ $10^{-3}$	Pa $\cdot$ s
Capacitance	$C_{1}$	1.0 $\times$ $10^{-9}$	F
Sensibility of optical lever	$g_{1}$	-4.0 $\times$ $10^{7}$	V/m

To realize self-excitation in a third-order system, the eigenvalues must be one negative real root and a pair of complex conjugate roots with a positive real part. According to the results shown in Fig. 12a, the self-excitation of the microcantilever in air begins when the resistance is set at 40.6 $\mathrm{k}\Omega$. Using the first equation in Eq. (8), we obtain ${\alpha }_{cr}=0.5603$ at the critical point with a negative real eigenvalue $\textit{q}$ and a pair of conjugate purely imaginary eigenvalues ${\pm {i\omega }}$. Substitution of $i\omega$ into Eq. (10) and separation of the real and imaginary parts yields

12 $$\begin{aligned} {\left\{ \begin{array}{ll} {\mathrm{Re}}: {\left( {\beta +\gamma +\frac{1}{{\alpha }_{cr}}}\right) }{{\omega }^{2}}-{\frac{1}{{\alpha }_{cr}}}=0, \\ {\mathrm{Im}}: {{\omega }^{3}}-{\left( {1-\frac{\beta -\gamma }{{\alpha }_{cr}}}\right) }{\omega }=0. \end{array}\right. } \end{aligned}$$

From Eq. (12), the relationship between ${\alpha }_{cr}$ and $\beta$ is calculated to be

13 $$\begin{aligned} {{\alpha }_{cr}}{{\beta }^{2}} + ({1-{{{\alpha }_{cr}}}^{2}}){{\beta }} - {\gamma }({{{{\alpha }_{cr}}}^{2}}+{{\alpha }_{cr}}{\gamma }+1) = 0. \end{aligned}$$

We then obtain $\beta$ = − 1.2366. From the second equation in Eq. (8), the piezoelectric coefficient ${g}_{3}$ is identified experimentally as 0.85 $\times$ $10^{-7}$ m/V, where the linear feedback ${g}_{2}$ is 5000 [1/s], in accordance with the experimental value.

**Figure 11:**
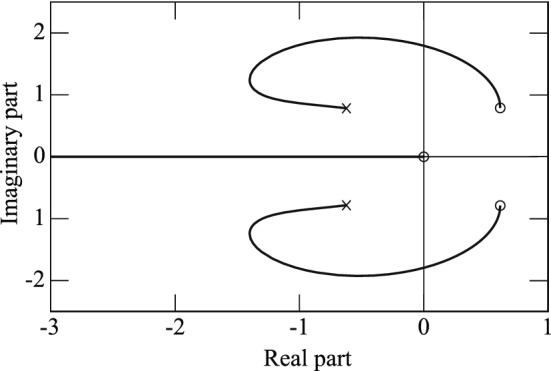
Root locus of the self-excited system.

Because the parameters for the entire self-excited system have been determined, the root locus produced by varying $\alpha$ is shown in Fig. 11, where we change $\alpha$ from 0 to $\infty$, which are represented by the symbols $\times$ and $\circ$, respectively. Meanwhile, the real part of a pair of complex conjugate roots can be changed from negative to positive but the real root remains negative. The required self-excitation can be produced by setting the resistance value *R* appropriately. However, it is predicted using linear theory that the amplitude will grow infinitely. The amplitude remains constant, as shown in the subsequent experiments. Because self-excitation with the steady-state amplitude is produced by the nonlinear damping term as a van der Pol oscillator^31^, the cause may be dependent on the nonlinear characteristics of the contact force between the microcantilever and the workpiece. Further theoretical discussions will form part of a future work.

### Experimental demonstration of self-excitation using phase difference

**Figure 12:**
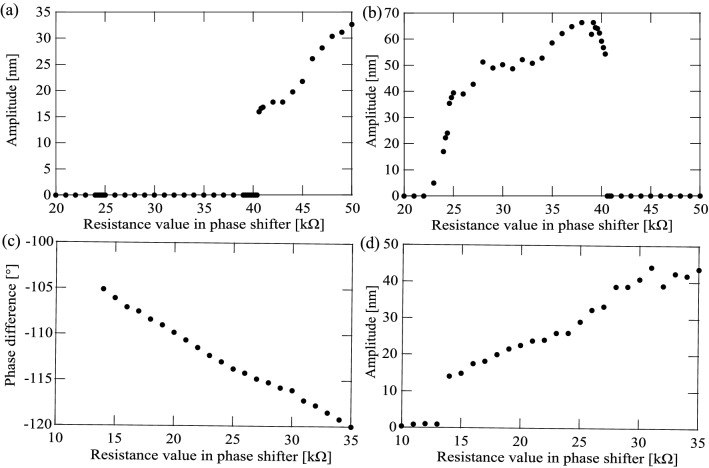
(**a**,**b**) show the amplitude magnitude changes in the cases when the base step and the microcantilever are self-excited in air, respectively, while the resistance value in the phase shifter is varied. (**c**,**d**) show change in the phase difference and the amplitude magnitude, respectively, caused by phase modulation under application of a 400 $\upmu$N pressing load while the resistance value in the phase shifter was varied from 10 $\mathrm{k}\Omega$ to 35 $\mathrm{k}\Omega$.

A method based on phase modulation is proposed for amplitude control of the self-excited microcantilever. In air, a constant linear feedback gain is set in advance. We then vary the resistance value *R* in the phase shifter from 20 $\mathrm{k}\Omega$ to 50 $\mathrm{k}\Omega$ to cause the phase difference between the input and the output of the self-excited electronic circuit to shift. When the resistance value is varied from 40.6 $\mathrm{k}\Omega$ to 50 $\mathrm{k}\Omega$, the response frequency is measured to change in the neighborhood of 13.03 kHz, which is almost agreement with the natural frequency of the base step 12.88 kHz, therefore we can conclude that only the base step is in the self-excited oscillation caused by appropriate setting of the phase difference, as shown in Fig. 12a. In the same way, the microcantilever can be self-excited individually when the resistance value is varied from 23 $\mathrm{k}\Omega$ to 40.4 $\mathrm{k}\Omega$, as shown in Fig. 12b, according to the response frequency measured to change in the neighborhood of 45.98 kHz, which is almost agreement with the natural frequency of the microcantilever 44.35 kHz.

Under application of a 400 $\upmu$N pressing load, we changed the resistance value in the phase shifter from 10 $\mathrm{k}\Omega$ to 35 $\mathrm{k}\Omega$ with a constant linear feedback gain. Figure 12c shows that the change in the resistance value causes the phase difference to shift. Figure 12d shows the change in the amplitude magnitude caused by the phase shift. Above 14 $\mathrm{k}\Omega$, self-excitation occurs with the single response frequency of 15.49 kHz, which is approximately equal to the natural frequency under the application of the 400 $\upmu$N pressing load, as shown in Fig. 4b. By shifting the phase difference, the amplitude can be varied arbitrarily under the 400 $\upmu$N pressing load to realize different hole cutting depths, as discussed in the next section. In addition, the hole cannot be made when the resistance value is zero. This result can be theoretically indicated from the root locus of Fig. 11 for the mathematical model. At $\alpha =0$ corresponding to $R=0\mathrm{k}\Omega$, because the three eigenvalues are a negative real and a complex conjugate with a negative value, the self-excite oscillation is not produced.

### Varying the cutting depth with a controlled amplitude using the phase modulation

**Figure 13:**
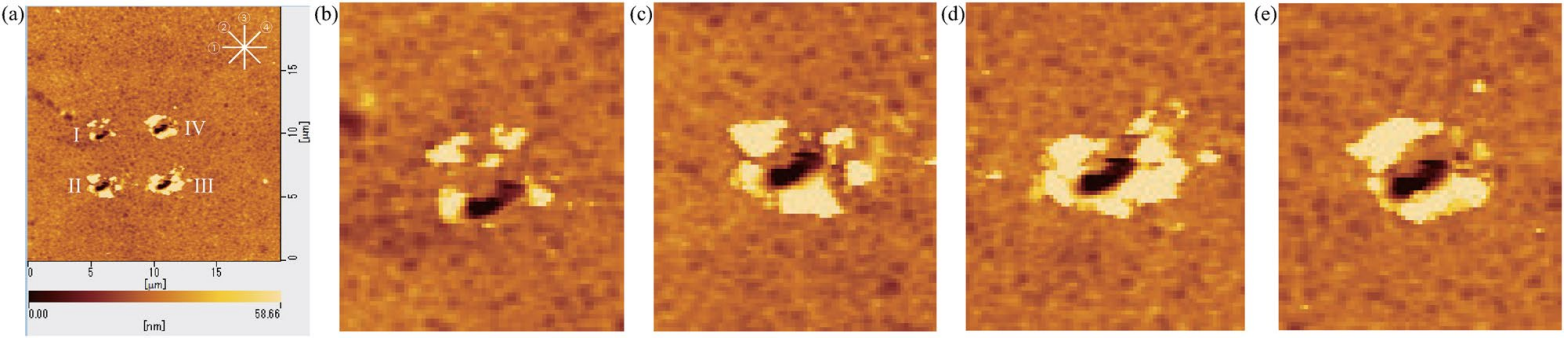
AFM image (**a**) shows four holes marked as I, II, III and IV which were cut under the 400 $\upmu \mathrm{N}$ pressing load by phase difference modulations with the self-excitation cutting method. The four holes were cut while the resistance values in the phase shifter circuit were set at 15 $\mathrm{k}\Omega$, 20 $\mathrm{k}\Omega$, 25 $\mathrm{k}\Omega$ and 30 $\mathrm{k}\Omega$, respectively. Four lines $\textcircled {1}$–$\textcircled {4}$ in the upper corner are to indicate the locations taken for the sectional views of four holes shown in Fig. 2 in Appendix C and the intersecting sectional views are centered on all holes. (**b**–**e**) show the magnified depiction for the AFM image of each hole.

**Figure 14:**
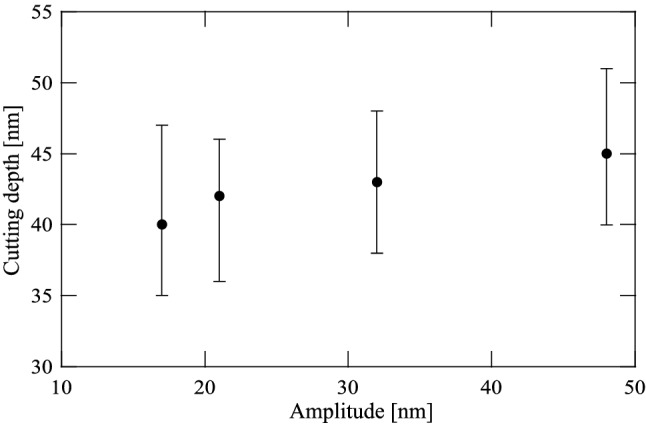
Depths for the holes I, II, III and IV are the average value for all sectional views for each hole. Four holes were cut under application of the 400 $\upmu \mathrm{N}$ pressing load when the four resistance values in the phase shifter were set as 15 $\mathrm{k}\Omega$, 20 $\mathrm{k}\Omega$, 25 $\mathrm{k}\Omega$ and 30 $\mathrm{k}\Omega$, respectively. The corresponding amplitudes were 17 nm, 21 nm, 32 nm and 48 nm, respectively.

In the cutting experiments performed under application of the 400 $\upmu \mathrm{N}$ pressing load, the four resistance values in the phase shifter were set as 15 $\mathrm{k}\Omega$, 20 $\mathrm{k}\Omega$, 25 $\mathrm{k}\Omega$ and 30 $\mathrm{k}\Omega$. The corresponding amplitudes were 17 nm, 21 nm, 32 nm and 48 nm, respectively. Four holes were cut and marked as I, II, III and IV with different magnitudes under the controlled amplitude condition, as shown in Fig. 13a. Four lines $\textcircled {1}$–$\textcircled {4}$ in the upper corner of Fig. 13a are to indicate the locations taken for the sectional views of four holes which are shown in Figs. 2a–d in Appendix C. The intersecting sectional views are centered on all cutting holes. Figure 13b–e show the magnified depiction for the AFM image of each cutting hole. The results indicate that deeper hole cutting is achieved because of the greater magnitude of the controlled amplitude, as illustrated in Fig. 14. It is thus demonstrated experimentally that holes can be cut with various depths under the 400 $\upmu \mathrm{N}$ pressing load with controlled amplitudes by varying the phase difference in the self-excitation cutting method.

## Conclusion

A self-excitation cutting method is proposed to improve the efficiency of the nanoscale cutting and the validity and usefulness of it are demonstrated through a series of cutting experiments. The deeper holes can be cut under application of the same pressing load than using the external excitation cutting method, because the microcantilever can be maintained in its resonant state independently of the pressing load when using the proposed method. Also, it is demonstrated experimentally that the amplitude of the microcantilever can be controlled to have different magnitudes by shifting the phase difference between the microcantilever deflection vibration signal and the feedback signal. The relationship between the amplitude and the hole cutting depth has been used to demonstrate that the deeper holes are cut at larger magnitudes of the controlled amplitude. The relationship between the response amplitude of the self-excited microcantilever and the phase shift are theoretically investigated. Together with this result, we will present the experimental scratching result depending on the amplitude of the microcantilever in a future research.

## Supplementary Information

Supplementary Information.
