# Supplementary material for: Nanoscale cutting using self-excited microcantilever
Source: Sci Rep. 2022 Jan 12;12:618. doi: 10.1038/s41598-021-04085-y (PMC8755816; doi:10.1038/s41598-021-04085-y)
Supplement: Supplementary file 1 — Supplementary Information. [file 41598_2021_4085_MOESM1_ESM.pdf]

Appendix A. Comparison of the amplitude magnitudes of the microcantilever in air and contact conditions.

An optical lever is used to measure the response amplitude. Then, we measure the angular displacement at the tip of the cantilever. It is theoretically shown that the angular displacement at the tip, where the boundary condition is simply supported (in contact), is greater than that, where the boundary condition is free (in air), as follows.

The equation of motion of the microcantilever is

$$EI\frac{\partial^4 v}{\partial x^4}+\rho A\frac{\partial^2 v}{\partial t^2}=0, \tag{1}$$

where E, I, ρ and A denote Young's modulus, cross-sectional secondary moment of inertia, density and cross-sectional area of the microcantilever, respectively. v is the lateral displacement of the microcantilever. x is the horizontal position coordinate whose origin is set at one end of the microcantilever. t is time.

As is well known, the lateral displacement v can be expressed as follows:

$$v(x,t)=h(x)g(t). \tag{2}$$

where $h(x)$ and $g(t)$ are governed by the following equations:

$$EI\frac{\mathrm{d}^4 h}{\mathrm{d}x^4}-c\rho A h(x)=0, \tag{3}$$

$$\frac{\mathrm{d}^2 g(t)}{\mathrm{d}t^2}+cg(t)=0, \tag{4}$$

The constants c are angular natural frequencies. The solution of Eq. (3) is expressed as:

$$h(x)=D_1\cos\beta x+D_2\sin\beta x+D_3\cosh\beta x+D_4\sinh\beta x, \tag{5}$$

where $\beta=\sqrt[4]{\frac{cEI}{\rho A}}$.

To compare the magnitudes of the angular displacements, $h(x)$ is normalized as $h^*(x)$ from 0 to l with respect to x. We obtain

$$h^*(x)=\frac{h(x)}{\sqrt{\int_0^l h^2(x)dx}}. \tag{6}$$

where l is the position of the tip of the microcantilever.

We consider two boundary conditions at the tip: (I) the free condition corresponding to the condition in air and (I I) simply supported condition corresponding to the condition with contact, i.e., $h(0)=0, \frac{\partial h(0)}{\partial x}=0, \frac{\partial^2 h(l)}{\partial x^2}=0, \frac{\partial^3 h(l)}{\partial x^3}=0$; $h(0)=0, h(l)=0, \frac{\partial h(0)}{\partial x}=0, \frac{\partial^2 h(l)}{\partial x^2}=0$. Therefore, in the conditions (I) and (II), the angular displacements of the microcantilever tip are approximately expressed at the tip, respectively as:

$$h^*(l)=\frac{2.75}{l^{\frac{3}{2}}}, \tag{7}$$

$$h^*(l)=\frac{-5.71}{l^{\frac{3}{2}}}. \tag{8}$$

From the results, it can be obtained that the angular displacement of the microcantilever tip under the boundary condition (I I) is greater than that under the boundary condition (I). This is one of explanations that the amplitude in Fig. 4(b) is lager than that in Fig. 4(a).

Appendix B. Sectional views of the cutting results with externally excited cutting method.

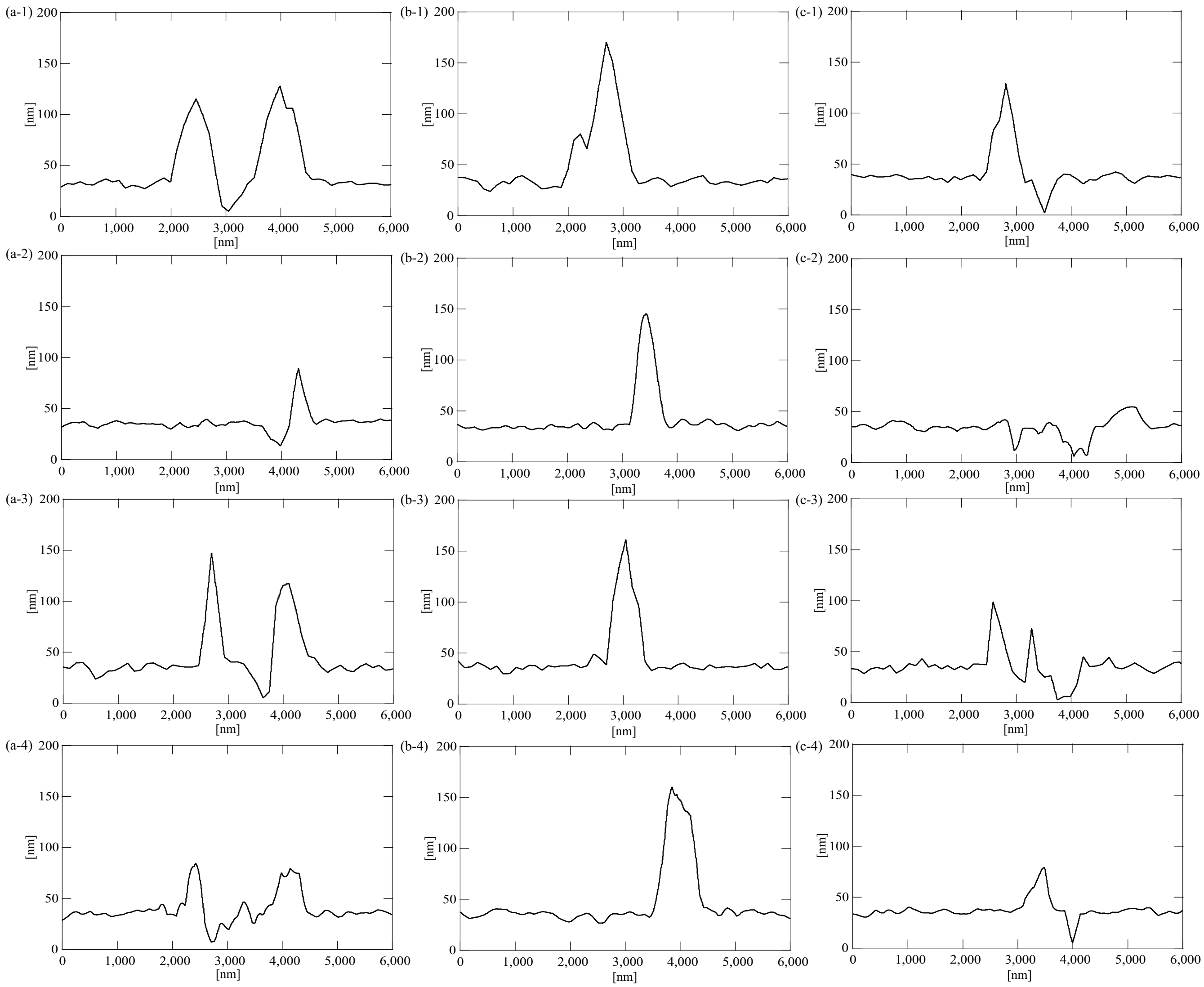

Figure 1. (a-1)-(a-4) represent the sectional views of the hole I in Fig. 5(b) obtained by four lines ①-④, respectively. (b-1)-(b-4) represent the sectional views of the cutting result Ⅱ in Fig. 5(b) obtained by four lines ①-④, respectively. (c-1)-(c-4) represent the sectional views of the hole Ⅲ in Fig. 5(b) obtained by four lines ①-④, respectively.

Table 1. Depth for the hole I calculated by each sectional view obtain by each line shown in Fig. 1.

Line	Depth(Max) [nm]	Depth(Min) [nm]	Depth(Average) [nm]
①	32	22	27
②	30	20	26
③	41	25	30
④	33	21	29

Table 2. Depth for the hole Ⅲ calculated by each sectional view obtain by each line shown in Fig. 1.

Line	Depth(Max) [nm]	Depth(Min) [nm]	Depth(Average) [nm]
①	40	28	34
②	35	24	29
③	41	26	32
④	35	24	31

Table 3. All parameters of the cutting conditions and results in three cutting experiments with the external excited cutting method. Depth for the holes I and Ⅲ is calculated in the average for all sectional views for each hole.

Hole	Pressing load [μN]	Excitation frequency [kHz]	Amplitude [nm]	Depth(Max) [nm]	Depth(Min) [nm]	Depth(Average) [nm]
I	200	15.41	26	34	22	28
Ⅱ	400	15.41	12	N/A	N/A	N/A
Ⅲ	400	15.49	32	38	27	32

Appendix C. Sectional views of the holes with self-excited cutting method.

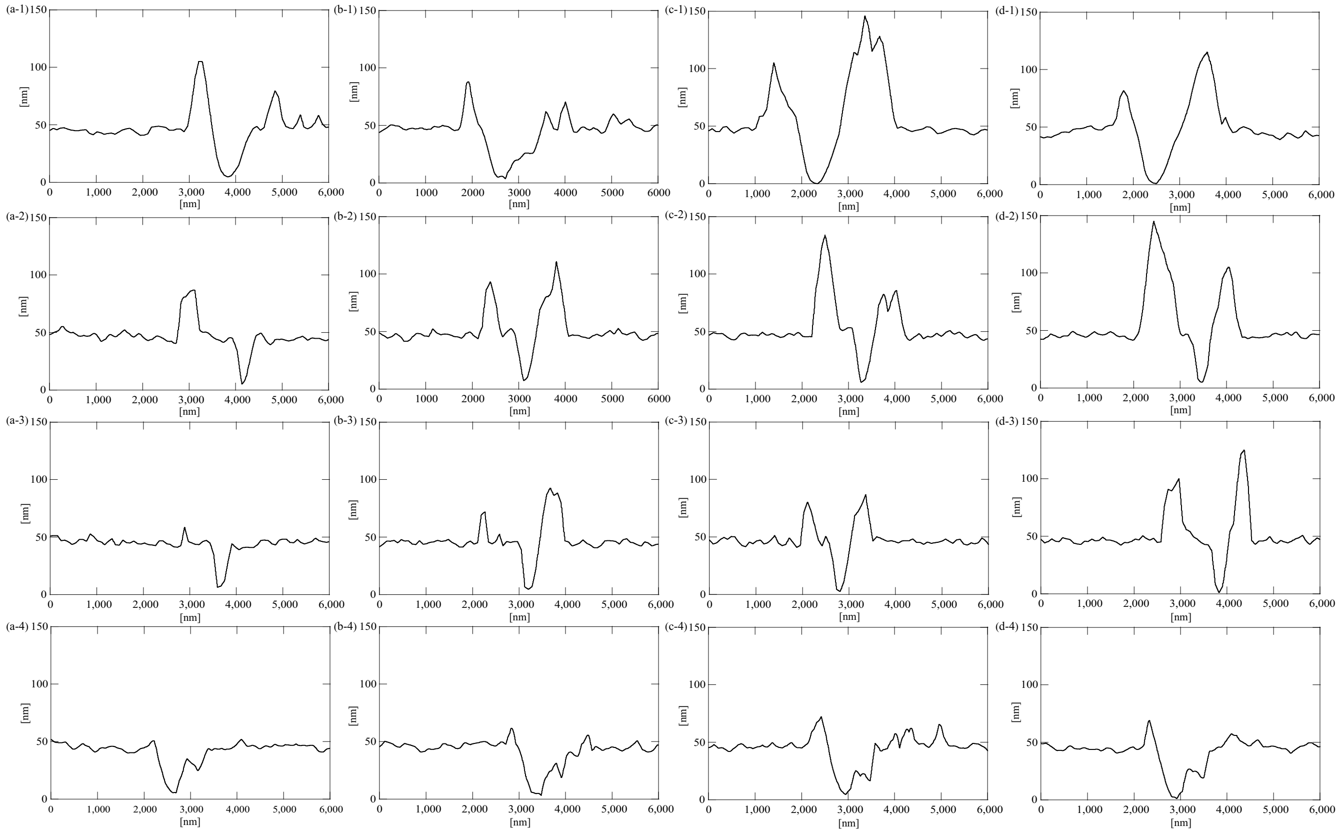

Figure 2. (a-1)-(a-4) represent the sectional views of the hole I in Fig. 13(b) obtained by four lines ①-④, respectively. (b-1)-(b-4) represent the sectional views of the hole Ⅱ in Fig. 13(b) obtained by four lines ①-④, respectively. (c-1)-(c-4) represent the sectional views of the hole Ⅲ in Fig. 13(b) obtained by four lines ①-④, respectively. (d-1)-(d-4) represent the sectional views of the hole Ⅳ in Fig. 13(b) obtained by four lines ①-④, respectively.
